# Participation of GABA_A_ Chloride Channels in the Anxiolytic-Like Effects of a Fatty Acid Mixture

**DOI:** 10.1155/2013/121794

**Published:** 2013-09-19

**Authors:** Juan Francisco Rodríguez-Landa, Rosa Isela García-Ríos, Jonathan Cueto-Escobedo, Blandina Bernal-Morales, Carlos M. Contreras

**Affiliations:** ^1^Laboratorio de Neurofarmacología, Instituto de Neuroetología, Universidad Veracruzana, 91190 Xalapa, VER, Mexico; ^2^Unidad Periférica Xalapa, Instituto de Investigaciones Biomédicas, Universidad Nacional Autónoma de México, 91190 Xalapa, VER, Mexico

## Abstract

Human amniotic fluid and a mixture of eight fatty acids (FAT-M) identified in this maternal fluid (C12:0, lauric acid, 0.9 **μ**g%; C14:0, myristic acid, 6.9 **μ**g%; C16:0, palmitic acid, 35.3 **μ**g%; C16:1, palmitoleic acid, 16.4 **μ**g%; C18:0, stearic acid, 8.5 **μ**g%; C18:1*cis*, oleic acid, 18.4 **μ**g%; C18:1*trans*, elaidic acid, 3.5 **μ**g%; C18:2, linoleic acid, 10.1 **μ**g%) produce anxiolytic-like effects that are comparable to diazepam in Wistar rats, suggesting the involvement of **γ**-aminobutyric acid-A (GABA_A_) receptors, a possibility not yet explored. Wistar rats were subjected to the defensive burying test, elevated plus maze, and open field test. In different groups, three GABA_A_ receptor antagonists were administered 30 min before FAT-M administration, including the competitive GABA binding antagonist bicuculline (1 mg/kg), GABA_A_ benzodiazepine antagonist flumazenil (5 mg/kg), and noncompetitive GABA_A_ chloride channel antagonist picrotoxin (1 mg/kg). The FAT-M exerted anxiolytic-like effects in the defensive burying test and elevated plus maze, without affecting locomotor activity in the open field test. The GABA_A_ antagonists alone did not produce significant changes in the behavioral tests. Picrotoxin but not bicuculline or flumazenil blocked the anxiolytic-like effect of the FAT-M. Based on the specific blocking action of picrotoxin on the effects of the FAT-M, we conclude that the FAT-M exerted its anxiolytic-like effects through GABA_A_ receptor chloride channels.

## 1. Introduction

The *γ*-aminobutyric acid-A (GABA_A_) receptor is a heteropentameric structure that consists of several subunits with GABA, benzodiazepine, alcohol, barbiturate, and neurosteroid recognition sites [1]. The activation of GABA_A_ receptors increases the intraneuronal concentration of chloride ions, leading to hyperpolarization, an action shared by anxiolytic, hypnotic, sedative, and anticonvulsant drugs [2]. Consequently, GABA_A_ receptors are considered the main target for clinically effective anxiolytic drugs and some neurosteroids with potential anxiolytic properties [3, 4].

Several GABA_A_ receptor antagonists, such as bicuculline, flumazenil, and picrotoxin, are currently used to identify the precise sites of action of drugs on the GABA_A_ receptor. These antagonists are able to block the anxiolytic-like effects of diazepam and some neurosteroids [1, 5, 6]. Their actions are well known. Bicuculline is a competitive antagonist of the GABA binding site and blocks the anxiolytic-like effects of 4′-chlorodiazepam and progesterone [7], among others. Flumazenil is a selective antagonist of the benzodiazepine allosteric binding site and blocks the anxiolytic-like effects of diazepam, alprazolam, and some neurosteroids [4, 8, 9]. Picrotoxin is a noncompetitive antagonist of GABA_A_ receptor chloride channels and blocks the anxiolytic-like effects of diazepam, 4′-chlorodiazepam, progesterone, and allopregnanolone [3, 7, 10, 11], among others. Therefore, these antagonists constitute effective tools in the pharmacological screening of drugs that interact with GABA_A_ receptors [12].

Recently it was demonstrated that human amniotic fluid and a mixture of eight fatty acids (FAT-M) contained therein produce anxiolytic-like effects similar to diazepam in male and female adult Wistar rats [13]. The FAT-M included eight FATs (C12:0, lauric acid; C14:0, myristic acid; C16:0, palmitic acid; C16:1, palmitoleic acid; C18:0, stearic acid; C18:1*cis*, oleic acid; C18:1*trans*, elaidic acid; C18:2, linoleic acid) that were consistently detected in human amniotic fluid, colostrum, and milk. In addition to its anxiolytic effects [13], the FAT-M produced appetitive responses in human newborns [14]. The anxiolytic-like action of the FAT-M may involve the participation of GABA_A_ receptors, given that some FATs modulate the opening of GABA_A_ receptor chloride channels in vitro [15], but this possibility needs to be assayed in vivo.

Therefore, the present study confirmed the anxiolytic-like effect of a FAT-M using two validated experimental animal models currently used to test the effectiveness of clinically effective anxiolytics, the defensive burying test [16] and elevated plus maze [17]. We then explored the GABA_A_ site of action involved in the anxiolytic-like effects of a FAT-M using bicuculline (a competitive antagonist of the GABA_A_ receptor), flumazenil (a blocker of the benzodiazepine recognition site), and picrotoxin (a noncompetitive antagonist of chloride channels).

## 2. Material and Methods

### 2.1. Ethics

All of the experimental procedures in the present study followed the principles of animal care based on the *Guide for the Care and Use of Laboratory Animals* (National Research Council, [18]). The protocol received authorization from the Biomedical Research Institute Ethical Committee (Universidad Nacional Autónoma de México).

### 2.2. Animals

Male Wistar rats were obtained from a local strain supplied by Harlan (México City, México). They were housed in local housing facilities at a mean temperature of 25 ± 2°C with a 12 h/12 h light/dark cycle (lights on at 7:00 AM). All of the rats included in the study were approximately 2 months old, weighed 250–300 g, and five to six rats were housed per cage in acrylic boxes (44 cm width × 33 cm length × 20 cm height) with ad libitum access to food (Teklad Lab Animal Diets; Harlan, Indianapolis, IN, USA) and purified water. All of the experiments were performed during the light period (approximately 12:00 PM).

### 2.3. Behavioral Tests

#### 2.3.1. Defensive Burying Test

An acrylic box (27 cm width × 17.5 cm length × 30 cm height) with the floor covered by a 5 cm bed of fine sawdust (Teklad Sani-Chips 7090, 2.2 cubic feet; Harlan, Indianapolis, IN, USA) was placed inside a noise-isolated box (65 cm width × 55 cm length × 45 cm height; Coulbourn Instruments, Whitehall, PA, USA). An electrode (7 cm length, 0.5 cm diameter) protruded 2 cm above the sawdust bed horizontally from one wall of the box (17.5 × 30 cm) [16, 19]. The electrode delivered a constant-intensity current (0.3 mA, direct current) through an electronic stimulator (Grass Instruments S44, Quincy, MA, USA) coupled in series to a stimulus isolation unit (Grass Instruments SIU5) and constant-current unit (Grass Instruments CCUIA). When a rat incidentally touched the electrode, it received an electric shock and began to vigorously displace the sawdust to cover the electrode (burying behavior). All of the sessions were recorded with a digital videocamera (Sony, DCR-SR85, 25x optical zoom, Carl Zeiss lens) for subsequent analysis by two independent observers to measure burying latency and total cumulative burying time during a 10 min test, starting from the first electric shock. After each test session, the bed of fine sawdust was removed and replaced by clean sawdust bedding. Only observations with more than 95% agreement between observers were included in the data analysis.

#### 2.3.2. Elevated Plus Maze

The apparatus was constructed of wood and situated in a brightly lit room (40 lux). The apparatus consisted of two opposite open and closed arms set in a perpendicular configuration. The open and closed arms were painted white and black, respectively. The dimensions of the open arms were 50 cm length × 10 cm width and the closed arms were 50 cm length × 10 cm width × 40 cm height. The entire maze was elevated 50 cm from the floor. The rats were placed in the center of the maze, facing an open arm, and the time spent on and number of entries into the open arms was recorded [20]. The total number of entries (open arms + closed arms) and percentage of open arm entries ((open entries)/(total entries) × 100) were calculated. The elevated plus maze was cleaned with a 5% ethanol solution after each session.

#### 2.3.3. Open Field Test

To evaluate the effects of the treatments on spontaneous locomotor activity, which could interfere with performance in the defensive burying test and elevated plus maze, the rats were subjected to a 5 min test in the open field after the defensive burying and elevated plus maze tests. We used an automated motor activity monitor (Acti-Track v2.7.10, PanLab, S.L. Instrument, Barcelona, Spain) in a Perspex box (45 × 45 cm base, 35 cm height). A total of 32 infrared beams, 16 each on perpendicular walls, were mounted 3 cm above the box frame floor and connected to an interface (LE 8811, LSI Letica Scientific Instruments, Barcelona, Spain), and the data were sent to a computer. For data analysis, the floor of the cage was virtually divided into five zones (four peripheral and one central). The total number of entries into the zones (i.e., crossings), time spent active, and time spent resting were recorded. Because of the relatively small cage, we did not compare central *versus* peripheral exploration.

After each experimental session, the open field was carefully cleaned and deodorized with a 5% ethanol cleaning solution. Five minutes elapsed between each test to allow the scent of the substances to dissipate.

### 2.4. Pretreatment

 The doses and pretreatment schedules were based on previous reports [4, 7, 21] that effectively antagonized the behavioral effects produced by anxiolytic drugs: bicuculline, 1 mg/kg; flumazenil, 5 mg/kg; picrotoxin, 1 mg/kg. The antagonists were administered intraperitoneally in an equivalent volume of 1 mL/kg 30 min before the FAT-M treatment, including the vehicles.

We used two vehicles, one for the GABA_A_ antagonists (vehicle-1: 1% Tween-80, 14% propylene glycol, and 85% saline) and another for the FAT-M (vehicle-2: 96% propylene glycol and 4% ethanol). All of the chemical compounds were purchased from Sigma-Aldrich (St. Louis, MO, USA).

### 2.5. Treatment

The FAT-M preparation and treatment schedule were based on previous reports [13]. The FAT-M consisted of lauric acid (0.4 mg), myristic acid (3.0 mg), palmitic acid (15.3 mg), palmitoleic acid (7.1 mg), stearic acid (3.7 mg), oleic acid (8.0 mg), elaidic acid (1.5 mg), and linoleic acid (4.4 mg) in 100 mL of vehicle-2 at a temperature <40°C. The FAT-M (1 mL/rat) or vehicle-2 was subcutaneously injected 60 min before the behavioral tests. Analytical-grade FATs were purchased from Sigma-Aldrich.

### 2.6. Experimental Groups

#### 2.6.1. Intrinsic Activity of GABA_A_ Antagonists

To identify the intrinsic activity of the GABA_A_ antagonists on anxiety-like behavior and open field activity, four independent groups were evaluated in the defensive burying test and subsequently open field test. The treatment conditions included four combinations: vehicle-1 before vehicle-2 (vehicles; *n* = 11), bicuculline before vehicle-2 (bicuculline; *n* = 12), flumazenil before vehicle-2 (flumazenil; *n* = 12), and picrotoxin before vehicle-2 (picrotoxin; *n* = 11). Other four independent groups (*n* = 8 rats per group) received a similar treatment schedule but were tested in the elevated plus maze and subsequently open field test.

#### 2.6.2. Interaction between GABA_A_ Antagonists and FAT-M

The defensive burying test included five independent groups that received five different combinations: vehicle-1 before vehicle-2 (vehicles; *n* = 11), vehicle-1 before FAT-M (FAT-M; *n* = 15), bicuculline before FAT-M (bicuculline + FAT-M; *n* = 11), flumazenil before FAT-M (flumazenil + FAT-M; *n* = 11), and picrotoxin before FAT-M (picrotoxin + FAT-M; *n* = 13). The elevated plus maze test included another five independent groups (*n* = 8 rats per group) that received similar treatments. The open field test was conducted less than 5 min after the elevated plus maze test.

### 2.7. Statistical Analysis

All of the data were statistically analyzed using one-way analysis of variance (ANOVA). Values of *P* ≤ 0.05 were followed by the Student-Newman-Keuls *post hoc*. We first analyzed the intrinsic activity of GABA_A_ antagonists on the variables in the behavioral tests. We then evaluated the effects of antagonism of different binding sites of the GABA_A_ receptor on the effects of the FAT-M. The results are expressed as mean ± standard error.

## 3. Results

### 3.1. Intrinsic Activity of GABA_A_ Antagonists

 The GABA_A_ antagonists alone did not affect burying latency (*F*_3,42_ = 0.46, *P* = 0.712) or cumulative burying time (*F*_3,42_ = 0.11, *P* = 0.958) in the defensive burying test. The antagonists also did not affect the number of crossings (*F*_3,42_ = 2.65, *P* = 0.061), activity time (*F*_3,42_ = 1.79, *P* = 0.164), or resting time (*F*_3,42_ = 2.33, *P* = 0.088) in the open field test. Likewise, the antagonists did not produce significant changes in the elevated plus maze, including the time spent in the open arms (*F*_3,28_ = 0.23, *P* = 0.875), number of entries into the open arms (*F*_3,28_ = 0.64, *P* = 0.592), total number of entries into the arms (*F*_3,28_ = 0.84, *P* = 0.483), and percentage of entries into the open arms (*F*_3,28_ = 0.88, *P* = 0.462). Similarly, no significant changes were found in the open field test, including the number of crossings (*F*_3,28_ = 1.61, *P* = 0.208), activity time (*F*_3,28_ = 2.19, *P* = 0.111), and resting time (*F*_3,28_ = 2.02, *P* = 0.134).

### 3.2. Interactions between GABA_A_ Antagonists and FAT-M

#### 3.2.1. Defensive Burying Test

**Figure 1:**
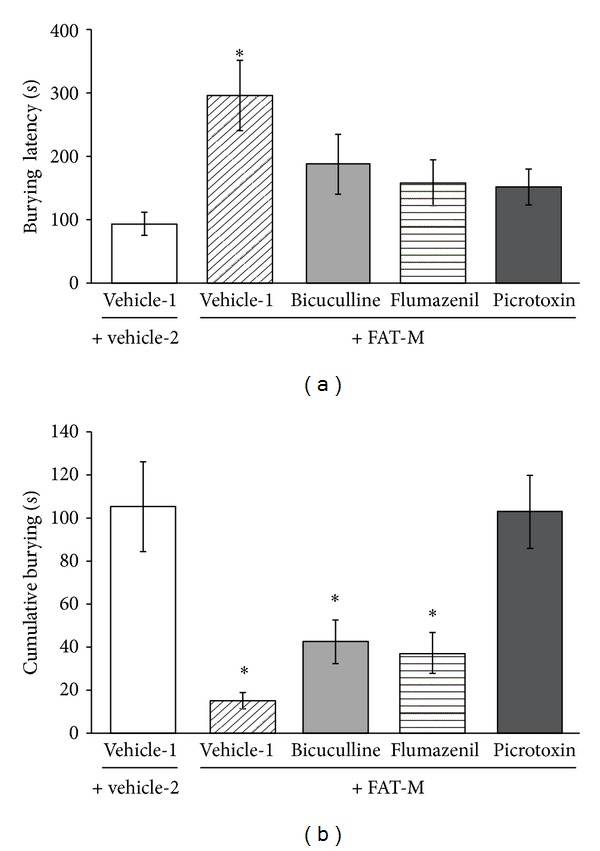
Defensive burying test. (a) Burying latency was significantly longer in the FAT-M group pretreated with vehicle-1, an effect attenuated by pretreatment with GABA_A_ antagonists. (b) Cumulative burying time was significantly shorter in the vehicle-1 + FAT-M, bicuculline + FAT-M, and flumazenil + FAT-M groups than in the vehicle-1 + vehicle-2, an effect not detected in the picrotoxin + FAT-M group (*P < 0.05, Student-Newman-Keuls *post hoc* test). FAT-M, fatty acid mixture.

The analysis of burying latency revealed significant differences between treatments (*F*_4,56_ = 3.45, *P* = 0.014). The *post hoc* test showed that burying latency was significantly longer in the FAT-M groups compared with the vehicle-1 + vehicle-2 group, but no significant differences were detected in the FAT-M groups pretreated with GABA_A_ antagonists compared with the vehicle-1 + vehicle-2 group (Figure 1(a)).

The analysis of cumulative burying time also revealed significant differences between treatments (*F*_4,56_ = 10.80, *P* = 0.001). The *post hoc* test revealed that cumulative burying time was significantly shorter in the FAT-M, bicuculline + FAT-M, and flumazenil + FAT-M groups than in the vehicle-1 + vehicle-2 group. However, the picrotoxin + FAT-M group was not significantly different from the vehicle-1 + vehicle-2 group (Figure 1(b)).

#### 3.2.2. Elevated Plus Maze

**Figure 2:**
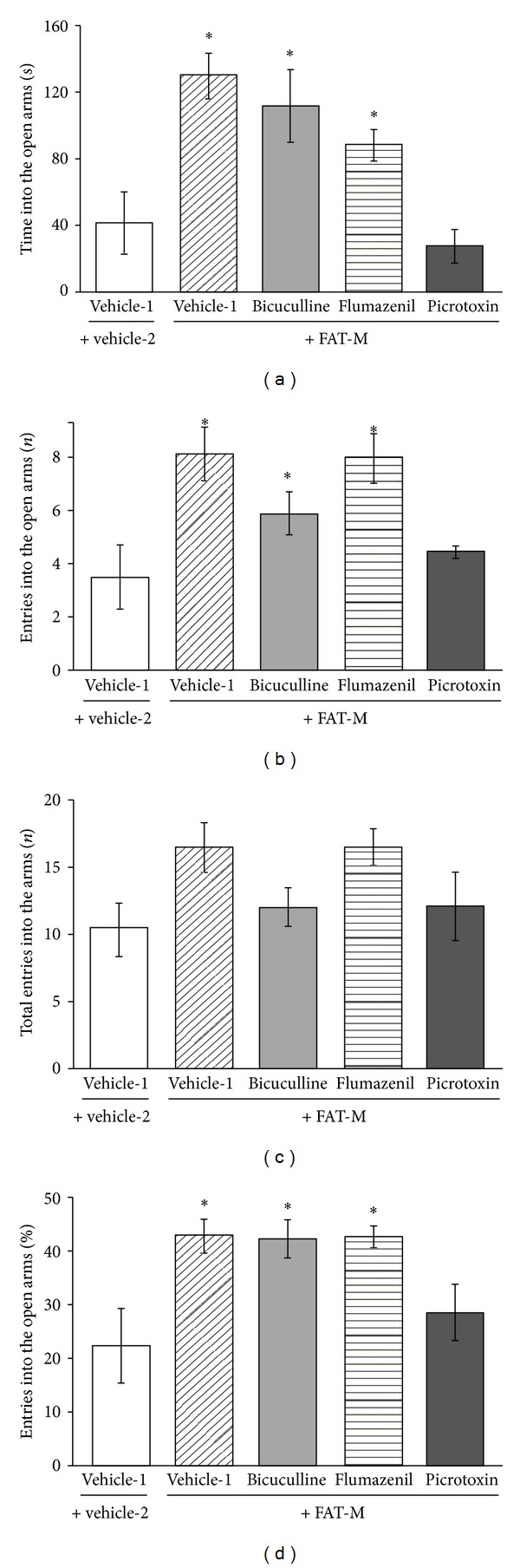
Elevated plus maze. The FAT-M produced anxiolytic-like effects that were blocked by pretreatment with picrotoxin. (a) Time spent in open arms. (b) Total number of entries into the arms. (c) Number of entries into the open arms. (d) Percentage of entries into the open arms. The elevated plus maze test lasted for 5 min. *P < 0.05, compared with vehicle-1 + vehicle-2 and picrotoxin + FAT-M groups (Student-Newman-Keuls *post hoc* test). FAT-M, fatty acid mixture.

The analysis of the time spent in the open arms revealed significant differences between treatments (*F*_4,35_ = 8.47, *P* = 0.001). The *post hoc* test revealed that the time spent in the open arms was longer in the FAT-M, bicuculline + FAT-M, and flumazenil + FAT-M groups than in the vehicle-1 + vehicle-2 and picrotoxin + FAT-M groups, which displayed similar values (Figure 2(a)). The number of entries into the open arms was also significantly different (*F*_4,35_ = 3.66, *P* = 0.014). The *post hoc* test revealed that this variable was higher in the FAT-M, bicuculline + FAT-M, and flumazenil + FAT-M groups than in the vehicle-1 + vehicle-2 and picrotoxin + FAT-M groups (Figure 2(b)), but no significant differences between these latter two groups were detected.

The analysis of the percentage of entries into the open arms also revealed significant differences (*F*_4,35_ = 4.54, *P* = 0.005). The percentage of entries into the open arms was greater in the FAT-M, bicuculline + FAT-M, and flumazenil + FAT-M groups than in the vehicle-1 + vehicle-2 and picrotoxin + FAT-M groups (Figure 2(d)). No significant differences were found between the vehicle-1 + vehicle-2 and picrotoxin + FAT-M groups. Finally, the total entries into the arms were not significantly different between groups (*F*_4,35_ = 2.54, *P* = 0.060; Figure 2(c)).

#### 3.2.3. Open Field Test

In the open field test, the analysis of the number of crossings did not reveal significant differences between treatments (*F*_4,35_ = 1.06, *P* = 0.391). Similarly, no significant differences were detected in activity time (*F*_4,35_ = 1.53, *P* = 0.213) or resting time (*F*_4,35_ = 1.65, *P* = 0.183; Table 1).

## 4. Discussion

The present study explored the participation of the GABA_A_ receptor complex in the anxiolytic-like effects of a mixture of eight FATs in male Wistar rats subjected to the defensive burying test and elevated plus maze. Pretreatment with picrotoxin but not bicuculline or flumazenil blocked the anxiolytic-like effects of FAT-M, without affecting behavior in the open field test.

In the defensive burying test, the time that elapsed between the first shock and first attempt at burying (i.e., burying latency) is inversely related to the rat's reactivity. The time spent burying (i.e., total cumulative burying) is an indicator of anxiety as discussed by Treit [16]. “Anxious” animals spend more time burying than animals treated with anxiolytic drugs, such as diazepam, which spend less time burying. In the present study, the FAT-M reduced cumulative burying time and increased burying latency, confirming its anxiolytic-like effects, as previously reported [13].

The elevated plus maze is widely used to explore anxiety-like behavior and the anxiogenic- or anxiolytic-like effects of drugs [20]. Anxiety-like behavior in the elevated plus maze is assumed when both the percentage of entries into and time spent on the open arms are reduced. The present results showed that the FAT-M increased both the percentage of entries into and time spent on the open arms, confirming an anxiolytic-like effect in a second test of anxiety. This anxiolytic-like effect was only blocked by picrotoxin and not by the other GABA_A_ receptor antagonists tested in the present study.

The present results suggest that GABA_A_ receptor chloride ion channels, but not benzodiazepine or GABA binding sites, participate in the anxiolytic-like effects of the FAT-M. Certainly, some FATs (i.e., oleic, linoleic, ricinoleic, and arachidonic acids) seemingly participate in the regulation of chloride ion channels [22]. Interestingly, oleic and linoleic acids are components of the FAT-M tested in the present study. Oleic acid increases the affinity of agonists for the benzodiazepine site of GABA_A_ receptors [23], thus modulating the opening of chloride channels. The modulation of ion channels by other FATs (e.g., myristic and arachidonic acids) occurs through indirect effects that involve their metabolic conversion to active oxygenated metabolites and other direct effects [24] by accumulating FATs in the phospholipid membrane bilayer and modifying membrane tension, leading to conformational changes in ion channels and altering ion conductance [25].

Chloride channels participate in the actions of substances with anxiolytic potency [26–28]. Other compounds that act on chloride channels produce similar anxiolytic-like effects as the FAT-M. Some endogenous steroids, as progesterone and allopregnanolone, are allosteric modulators of GABA_A_ chloride ion channels [29–32]. At physiological (i.e., nanomolar) concentrations, some steroids with a reduced A-ring promote channel opening frequency and increase chloride flux [28, 33, 34]. Majewska [28] demonstrated that steroids interact at the membrane protein and lipid interface, leading to an increased frequency of chloride channel opening. In fact, the actions of neurosteroids resemble the actions of flunitrazepam, muscimol, and pentobarbital on chloride channel function and are blocked by the noncompetitive GABA antagonist picrotoxin [26], suggesting some similarity between neurosteroids and the FAT-M, given that they share common actions, such as anxiolytic-like effects in experimental models of anxiety that can be blocked by picrotoxin.

Finally, some drugs are able to produce nonspecific changes in spontaneous locomotor activity that may interfere with performance in the defensive burying test and elevated plus maze. The open field test was conducted after the aforementioned behavioral tests, allowing us to exclude possible nonspecific locomotor effects of antagonists or FAT treatments. In the open field test, we found that none of the GABA_A_ antagonists alone or the FAT-M at the doses tested produced any significant changes on motor activity, similar to previous reports [4, 11, 13, 35]. Additionally, the dose administered of bicuculline and picrotoxin did not produce behaviour linked to seizures (i.e., facial/ear twitching, myoclonic jerks, among others), which are detected with higher doses than those used in the present study. Therefore, the anxiolytic-like effect of the FAT-M and blockade of this effect by GABA_A_ antagonists do not appear to be associated with nonspecific effects of the treatments on spontaneous locomotion.

A possible limitation of the present study was that the FAT-M was administered in addition to the rats' normal diet (i.e., purine), possibly providing an additional source of energy that may impact spontaneous behavior. However, we did not observe any change in locomotion associated with the FAT-M treatment, similar to previous reports [13, 36]. Although we did not directly assess the increased metabolic sources provided by the FATs, this may be considered inconsequential for interpreting the present results.

In conclusion, the anxiolytic-like effects of the FAT-M involved actions on GABA_A_ receptors, specifically chloride channels, providing additional evidence of the anxiolytic-like effects of FATs and the possible site of action on GABA_A_ receptor of the FAT-M used in the present study. However, the participation of other receptors linked to chloride ion channel (i.e., strychnine-sensitive glycine receptors) could not to be discarded, which needs to be explored in particular studies.

## Figures and Tables

**Table 1:** Open field test.

Group	Crossings	Activity time (s)	Resting time (s)
Vehicle-1 + Vehicle-2	21.8 ± 2.88	75.1 ± 7.05	221.9 ± 7.09
Vehicle-1 + FAT-M	37.5 ± 5.37	109.7 ± 8.82	194.8 ± 10.42
Bicuculline + FAT-M	36.0 ± 6.33	103.9 ± 8.73	201.3 ± 7.00
Flumazenil + FAT-M	38.1 ± 8.70	97.2 ± 14.56	184.0 ± 16.67
Picrotoxin + FAT-M	34.0 ± 7.63	90.3 ± 13.03	213.5 ± 13.87

No significant differences were found in the evaluated variables. The data are expressed as mean ± standard error. FAT-M, fatty acid mixture.
